# Diagnosis of Acute Glaucoma Attack Using a Quantitative Pupillometer: A Case Report

**DOI:** 10.7759/cureus.72850

**Published:** 2024-11-01

**Authors:** Yuta Koshikawa, Ayuko Sato, Ryo Umeda, Ryo Ichibayashi

**Affiliations:** 1 Department of Internal Medicine, Toho University Sakura Medical Center, Chiba, JPN; 2 Department of Orthopedic Surgery, Toho University Sakura Medical Center, Chiba, JPN; 3 Department of Internal Medicine/Division of Emergency Medicine, Toho University Sakura Medical Center, Chiba, JPN

**Keywords:** acute glaucoma attack, intraocular pressure, neurological pupil index, penlight method, pupillometer

## Abstract

Acute glaucoma attacks cause a sudden increase in intraocular pressure. In addition to ocular symptoms such as redness, visual impairment, and eye pain, it also presents with headache and vomiting. It is diagnosed using a slit lamp microscope, gonioscope, and anterior segment optical coherence tomography. A 72-year-old man visited the emergency room complaining of repeated vomiting and a throbbing headache in the right temporal region. Conjunctival congestion of the right was observed. However, this was not considered important at the initial examination stage because he had been diagnosed with a dry eye at a local ophthalmology clinic. No intracranial bleeding was found on a head CT scan. Glaucoma was suspected and diagnosed based on the difference between the left and right neurological pupil index (NPi) and miosis rate (CH) measured by quantitative pupillometry. Quantitative pupillometry is a simple examination method not dependent on the examiner's skill. If a difference between the left and right NPi and CH measured by quantitative pupillometry is observed in patients with headaches, it can help diagnose acute glaucoma attacks.

## Introduction

Acute glaucoma attack is an ophthalmic emergency characterized by a sudden increase in intraocular pressure, which poses a high risk of blindness. Symptoms include severe unilateral periocular pain, blurred vision, nausea or vomiting, and signs of ocular congestion [1]. Accurate diagnosis of glaucoma requires measuring intraocular pressure, stereoscopic nerve testing, and formal visual field testing [2].

This disease may not include eye pain or decreased vision, and it is often misdiagnosed as migraine [3]. In addition, an ophthalmologist must examine the pupils with a stationary device to diagnose this disease. Non-specialist physicians primarily use the subjective penlight test to diagnose the condition based on symptoms, signs, and pupillary findings. However, it is not easy for non-ophthalmologists to determine whether glaucoma exists. For this reason, pupillary assessment, such as pupil dilation and loss of light reflex, is important in addition to symptoms and signs [4].

To the best of our knowledge, there are no reports on the use of quantitative pupillometry to diagnose acute glaucoma attacks. We used quantitative pupillometry in the emergency department. Although pupil dilation was not observed, differences in other parameters were observed between the left and right eyes. We report a case in which the results of this study led to a diagnosis of acute glaucoma attack.

## Case presentation

**Figure 1:**
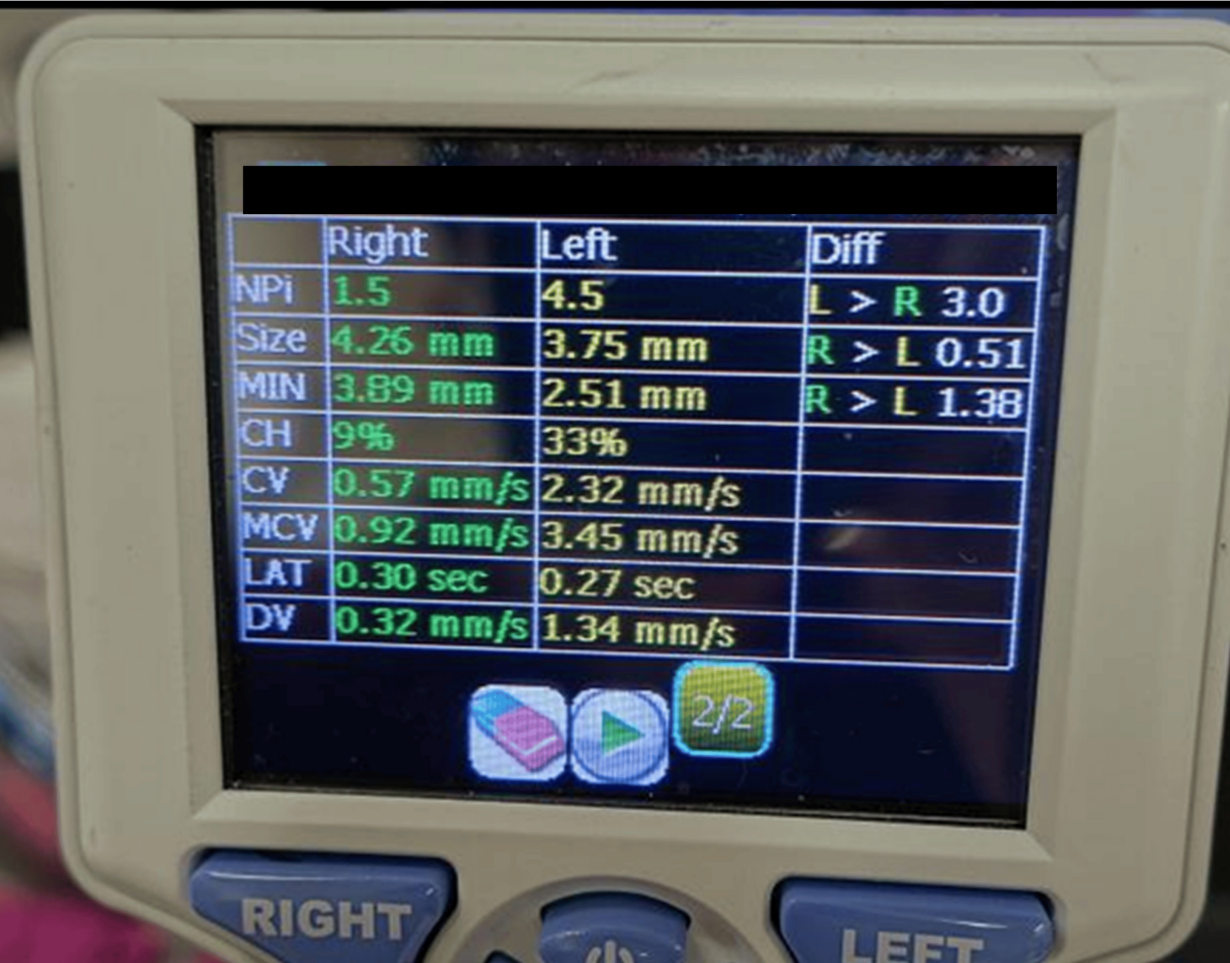
Data of quantitative pupillometer during acute glaucoma attacks. No pupil dilation was observed in the right eye, but the pupil contraction rate and speed were decreased compared to the left eye. NPi: neurological pupil index; SIZE: maximum pupil diameter; MIN: minimum pupil diameter; CH: pupillary constriction rate; CV: mean constriction velocity; MCV: maximum constriction velocity; LAT: reaction time; DV: mean dilation velocity

The patient was a 72-year-old man. His medical history included prostate cancer, cataracts, hypertension, and carotid artery stenosis. He was taking irbesartan, amlodipine, aspirin, lansoprazole, and lemborexant. He had been diagnosed with depression 10 days before his visit and had been taking escitalopram. One week before he visited our hospital, he complained of discomfort when opening his eyes and visited a local ophthalmologist. He was diagnosed with dry eye and prescribed hyaluronic acid eye drops, but his symptoms did not improve. On the day of the visit, he experienced eye congestion, vomiting, and a throbbing headache in the right temporal region, so he visited the emergency room. At the time of his visit, his level of consciousness was clear; his body temperature was 36.5°C, his respiratory rate was 15 breaths/minute, his pulse was 72 breaths/minute, and his blood pressure was 138/74 mmHg. Conjunctival congestion was observed in the right eye. Quantitative pupillometry did not reveal pupil dilation, but the right pupil was 0.51 mm larger than the left, and the pupillary reflex in the right eye was delayed and had a low constriction rate (Figure 1).

After quantitative pupillometry, the pupils were observed using the penlight method, but no difference in pupillary diameter was detected between the left and right eyes. However, the pupillary reflex was delayed in the right eye. No visual field defects were observed, and no other neurological abnormalities were found. No abnormalities were found on the head CT. The pounding score was 3 points.

**Figure 2:**
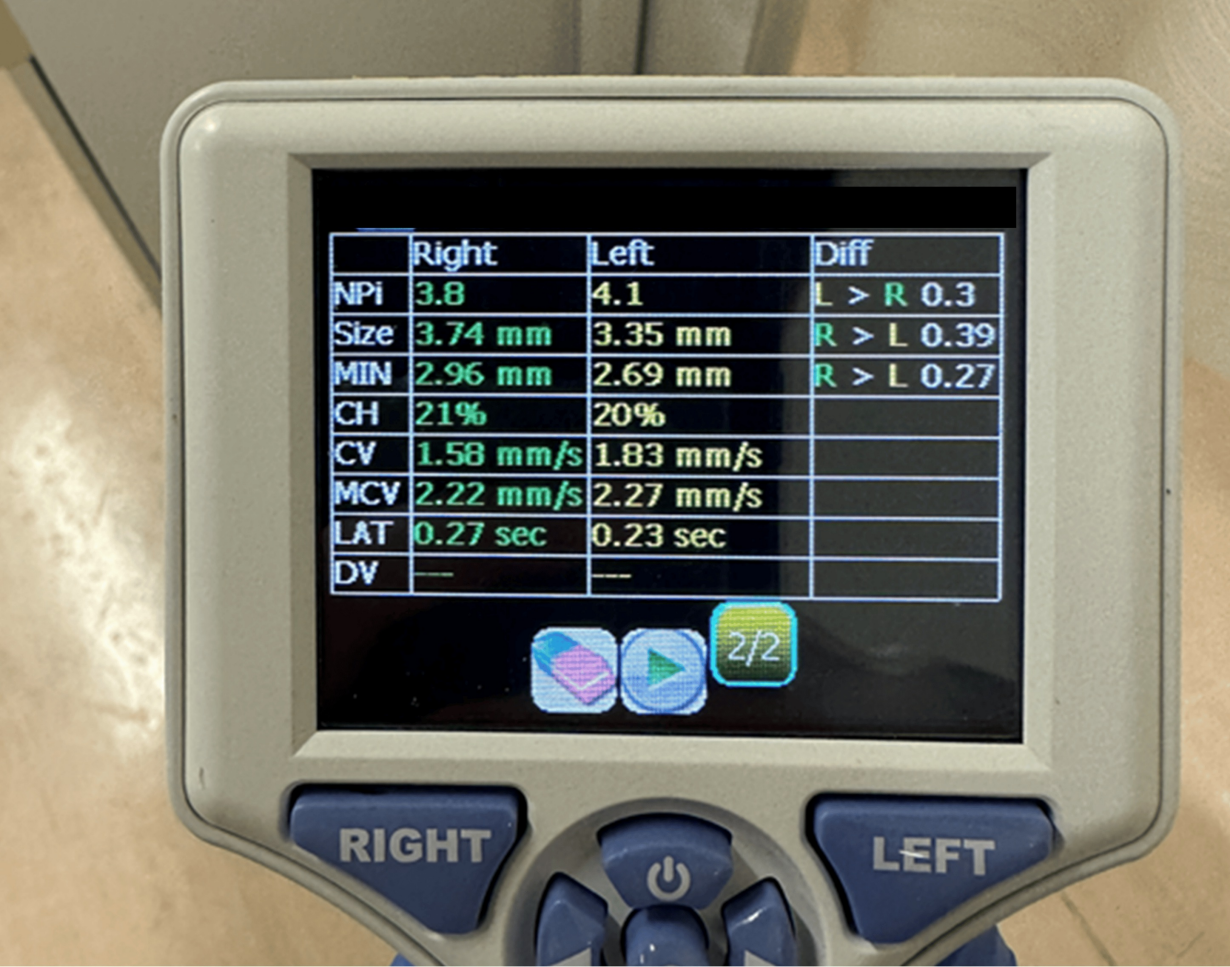
Data of quantitative pupillometer after treatment. There was no difference in pupil constriction rate or speed between the left and right eyes. NPi: neurological pupil index; SIZE: maximum pupil diameter; MIN: minimum pupil diameter; CH: pupillary constriction rate; CV: mean constriction velocity; MCV: maximum constriction velocity; LAT: reaction time; DV: mean dilation velocity

Upon repeat medical history, he noted decreased vision over the past seven days. Based on symptoms, signs, clinical course, and pupillary findings, an acute glaucoma attack was suspected, and an ophthalmologist was consulted. Examination by an ophthalmologist revealed ciliary congestion of the right bulbar conjunctiva, corneal edema, shallow anterior chamber, and narrow angle. Visual acuity was reduced to 6/60 in the right eye and 6/20 in the left eye. Intraocular pressure was 62 mmHg in the right eye and 7.5 mmHg in the left, with the right eye showing a marked increase in intraocular pressure. Based on these findings, an acute glaucoma attack was diagnosed, and treatment with frequent instillation of pilocarpine eye drops and mannitol infusion was started. 49 days after treatment, the symptoms disappeared, and data from quantitative pupillometry showed that the pupil constriction rate in the right eye improved and that the difference between the left and right pupils disappeared (Figure 2, Table 1).

**Table 1:** Data from quantitative pupillometry before and after treatment Although no pupil dilation was observed, CH, CV, and MCV decreased in the right eye before treatment, indicating miosis insufficiency. As a result of these factors, NPi decreased. After treatment, miosis insufficiency improved, and NPi also improved. NPi: neurological pupil index; SIZE: maximum pupil diameter; MIN: minimum pupil diameter; CH: pupillary constriction rate; CV: mean constriction velocity; MCV: maximum constriction velocity; LAT: reaction time; DV: mean dilation velocity

	Before treatment			After treatment			
	Right	Left	Difference	Right	Left	Difference	Reference range
NPi	1.5	4.5	L>R 3.0	3.8	4.2	L>R 0.3	3.0-4.9
SIZE (mm)	4.26	3.75	R>L 0.51	3.74	3.35	R>L 0.39	3.0-5.0
MIN (mm)	3.89	2.51	R>L 1.38	2.96	2.69	R>L 0.27	1.0-2.5
CH (％)	9	33	‐	21	20	‐	10-30
CV (mm/s)	0.57	2.32	‐	1.58	1.83	‐	0.5-2.5
MCV (mm/s)	0.92	3.45	‐	2.22	2.27	‐	2.5-6.0
LAT (sec)	0.3	0.27	‐	0.27	0.23	‐	<0.5
DV (mm/s)	0.32	1.34	‐	‐	‐	‐	0.3-1.0

The intraocular pressure also improved to 11 mmHg on the right and 8 mmHg on the left.

## Discussion

Frequent ocular symptoms of acute glaucoma attack include blurred vision (86%) and periocular pain (83%) [5]. Since these symptoms do not always appear, brain disease is often suspected. According to a single-center study of patients with acute glaucoma attack, 28% visited a specialist other than ophthalmologists, 32% underwent head imaging, and 8% underwent lumbar puncture [6].

The patient complained of headache and vomiting but no eye symptoms other than eye discomfort. For this reason, cerebral hemorrhage and migraine were initially suspected, but these were ruled out by head CT and Pounding score results [7]. Quantitative pupillometry uses light stimulation to measure maximum pupil diameter (SIZE), minimum pupil diameter (MIN), pupillary constriction rate (CH), mean constriction velocity (CV), maximum constriction velocity (MCV), reaction time (LAT), and mean dilation velocity (DV). In addition, a unique algorithm can be used to calculate the neurological pupil index (NPi), which calculates the speed of the light reflex based on these seven items. An NPi of 3.0 or higher is normal; the closer to 5.0, the quicker the reflex. Quantitative pupillometry revealed a difference in pupil NPi, CH, CV, and DV between the left and right pupils. Taking this opportunity, acute angle-closure glaucoma was suspected. In addition to a history of cataracts, selective serotonin reuptake inhibitors (SSRIs) are known to increase the risk of acute glaucoma attacks, and the initiation of oral administration of escitalopram may have triggered the attacks [1,8].

Traditionally, non-ophthalmologists have mainly diagnosed acute angle-closure glaucoma by checking for conjunctival congestion, corneal edema, pupil dilation on the affected side due to pupillary sphincter incompetence, and delayed light reflex using the penlight method. However, this method has variability in measurement results depending on the examiner and may lack accuracy [9-11]. There is also a method of checking intraocular pressure by transpalpebral palpation. Still, it is difficult to judge unless the intraocular pressure is high, at 70 mmHg or higher, compared with the average intraocular pressure of 52 mmHg [5]. These methods rely on subjective indicators, and doctors with little clinical experience in ophthalmology are expected to have difficulty making an accurate assessment.

In this case, the decrease in pupillary reflex velocity was quantitatively confirmed by the 0.51 mm difference between the left and right pupils in SIZE and the decrease in CV, MCV, CH, and NPi. The doctor in charge of this case had little clinical experience in ophthalmology and had never examined acute glaucoma attacks. However, he suspected this condition based on the above findings and requested an ophthalmologist to examine the patient, which enabled the diagnosis of acute angle-closure glaucoma. With the penlight method, it is difficult to determine the 0.51 mm difference between the left and right pupils or other decreases in reflex velocity unless the condition is severe. For this reason, there was a possibility that the diagnosis would not be made in the emergency department.

The symptoms subsequently disappeared after treatment at an ophthalmology clinic. When the pupils were observed again using a quantitative pupillometer, the pupillary reaction rate and NPi all improved. This suggests that non-ophthalmologists also may use the device to evaluate treatment progress for acute glaucoma attacks.

This way, the quantitative pupillometer does not depend on the examiner's skill and can quickly evaluate pupil findings. The strong point of the quantitative pupillometer is that it not only measures pupil size but also quantifies and visualizes pupil contraction rate and speed. In this case, it is convenient when non-ophthalmologists examine ophthalmic emergency cases in the emergency room, and it is considered to be a superior examination method compared to the existing penlight method. The quantitative pupillometer has been reported to be used in intensive care to evaluate the degree of brainstem ischemia in patients with acute brain injury and to evaluate neurological prognosis after cardiopulmonary arrest and resuscitation [12-14]. However, to the best of our knowledge, there have been no reports on diagnosing acute glaucoma attacks. For this reason, the widespread use of the quantitative pupillometer is thought to help prevent non-ophthalmologists from overlooking acute glaucoma attacks and diagnosing it. It may help reduce the risk of blindness. It is hoped that more cases will be accumulated in the future.

## Conclusions

Acute glaucoma attacks are sometimes misdiagnosed as migraines and are, therefore, often detected late, increasing the risk of blindness. However, they should always be considered when patients complain of headaches or vomiting. Evaluating ocular findings using a quantitative pupillometer can be a helpful evaluation device for doctors with little clinical ophthalmological experience when suspecting acute glaucoma attacks.

## References

[1] Ah-Kee EY, Egong E, Shafi A, Lim LT, Yim JL (2015). A review of drug-induced acute angle closure glaucoma for non-ophthalmologists. Qatar Med J.

[2] Gupta D, Chen PP (2016). Glaucoma. Am Fam Physician.

[3] Stan C, Stan C, Rednik AM (2020). Migraine or acute angle closure?. Rom J Ophthalmol.

[4] Kim JM, Park KH, Han SY, Kim KS, Kim DM, Kim TW, Caprioli J (2012). Changes in intraocular pressure after pharmacologic pupil dilation. BMC Ophthalmol.

[5] Foster PJ, Buhrmann R, Quigley HA, Johnson GJ (2002). The definition and classification of glaucoma in prevalence surveys. Br J Ophthalmol.

[6] Chua PY, Day AC, Lai KL (2018). The incidence of acute angle closure in Scotland: a prospective surveillance study. Br J Ophthalmol.

[7] Tejero Mas M, Burgos Blanco R, Gato Núñez C, Rivera Jiménez N, Aguirre Sánchez JJ, Buitrago F (2019). Validity and applicability of the mnemonic POUNDing rule in patients with migraine. A descriptive study [Article in Spanish]. Semergen.

[8] Chen VC, Ng MH, Chiu WC (2017). Effects of selective serotonin reuptake inhibitors on glaucoma: a nationwide population-based study. PLoS One.

[9] Nuessle S, Luebke J, Boehringer D, Reinhard T, Anton A (2022). Acute angle closure : An ophthalmological emergency in the emergency room [Article in German]. Med Klin Intensivmed Notfmed.

[10] Couret D, Boumaza D, Grisotto C (2016). Reliability of standard pupillometry practice in neurocritical care: an observational, double-blinded study. Crit Care.

[11] Taylor WR, Chen JW, Meltzer H (2003). Quantitative pupillometry, a new technology: normative data and preliminary observations in patients with acute head injury. Technical note. J Neurosurg.

[12] Du R, Meeker M, Bacchetti P, Larson MD, Holland MC, Manley GT (2005). Evaluation of the portable infrared pupillometer. Neurosurgery.

[13] Ritter AM, Muizelaar JP, Barnes T, Choi S, Fatouros P, Ward J, Bullock MR (1999). Brain stem blood flow, pupillary response, and outcome in patients with severe head injuries. Neurosurgery.

[14] Minami Y, Mishima S, Oda J (2020). Prediction of the level of consciousness using pupillometer measurements in patients with impaired consciousness brought to the emergency and critical care center. Acute Med Surg.

